# DNA Methylation Aberrations in Dimethylarsinic Acid-Induced Bladder Carcinogenesis

**DOI:** 10.3390/cancers15215274

**Published:** 2023-11-03

**Authors:** Tomoki Yamamoto, Min Gi, Satoshi Yamashita, Shugo Suzuki, Masaki Fujioka, Arpamas Vachiraarunwong, Runjie Guo, Guiyu Qiu, Anna Kakehashi, Minoru Kato, Junji Uchida, Hideki Wanibuchi

**Affiliations:** 1 Department of Molecular Pathology, Osaka Metropolitan University Graduate School of Medicine, 1-4-3 Asahi-machi, Abeno-ku, Osaka 545-8585, Osaka, Japan; d20mb037@st.osaka-cu.ac.jp (T.Y.);; 2 Department of Molecular Urology, Osaka Metropolitan University Graduate School of Medicine, 1-4-3 Asahi-machi, Abeno-ku, Osaka 545-8585, Osaka, Japan; 3 Department of Environmental Risk Assessment, Osaka Metropolitan University Graduate School of Medicine, 1-4-3 Asahi-machi, Abeno-ku, Osaka 545-8585, Osaka, Japan; 4 Department of Life Engineering, Faculty of Engineering, Maebashi Institute of Technology, 460-1 Kamisadori, Maebashi 371-0816, Gunma, Japan

**Keywords:** dimethylarsinic acid, bladder cancer, aberrant DNA methylation, CPXM1

## Abstract

**Simple Summary:**

Arsenic is a known carcinogen for the human urinary bladder. The present study explored aberrant DNA methylation in rat bladder carcinogenesis induced by dimethylarsinic acid (DMA^V^), a primary arsenic metabolite. Genome-wide DNA methylation and microarray gene expression analyses of DMA^V^-induced rat urothelial carcinoma (UC) and the urothelium of rats treated with DMA^V^ for 4 weeks identified 40 genes that were both hypermethylated and downregulated in DMA^V^-induced rat urothelial carcinoma. Notably, four of these genes (CPXM1, OPCML, TBX20, and KCND3) also showed reduced expression in the bladder urothelium after 4 weeks of exposure to DMA^V^. Furthermore, CPXM1 is aberrantly methylated and downregulated in human bladder cancers and human bladder cancer cells. Our findings highlight the significance of aberrant DNA methylation in DMA^V^-induced bladder carcinogenesis, implying early-stage arsenic-induced methylation changes.

**Abstract:**

Arsenic is a known human urinary bladder carcinogen. While arsenic is known to cause aberrant DNA methylation, the mechanism of arsenic-triggered bladder carcinogenesis is not fully understood. The goal of this study was to identify aberrant DNA methylation in rat bladder urothelial carcinoma (UC) induced by dimethylarsinic acid (DMA^V^), a major organic metabolite of arsenic. We performed genome-wide DNA methylation and microarray gene expression analyses of DMA^V^-induced rat UCs and the urothelium of rats treated for 4 weeks with DMA^V^. We identified 40 genes that were both hypermethylated and downregulated in DMA^V^-induced rat UCs. Notably, four genes (CPXM1, OPCML, TBX20, and KCND3) also showed reduced expression in the bladder urothelium after 4 weeks of exposure to DMA^V^. We also found that CPXM1 is aberrantly methylated and downregulated in human bladder cancers and human bladder cancer cells. Genes with aberrant DNA methylation and downregulated expression in DMA^V^-exposed bladder urothelium and in DMA^V^-induced UCs in rats, suggest that these alterations occurred in the early stages of arsenic-induced bladder carcinogenesis. Further study to evaluate the functions of these genes will advance our understanding of the role of aberrant DNA methylation in arsenic bladder carcinogenesis, and will also facilitate the identification of new therapeutic targets for arsenic-related bladder cancers.

## 1. Introduction

Arsenic is a widely recognized human carcinogen, and both environmental and industrial exposure has been associated with the development of urinary bladder cancers in humans [1]. The International Agency for Research on Cancer (IARC) classifies arsenic and inorganic arsenic compounds as Group 1 carcinogens [1]. Despite its known cancer risks, chronic arsenic exposure remains a substantial public health concern in various regions of the world. A comprehensive understanding of arsenic’s carcinogenic mechanisms can improve cancer risk assessment and contribute to developing effective therapeutic strategies.

A second and less well-known property of arsenic is its use in cancer therapy. Arsenic trioxide has demonstrated therapeutic efficacy in the treatment of acute promyelocytic leukemia [2]. Studies have also indicated its potential effectiveness in treating certain solid tumors [3,4]. For the interested reader, there are currently two active (NCT01409161: Tretinoin and Arsenic Trioxide with or without Gemtuzumab Ozogamicin in Treating Patients with Previously Untreated Acute Promyelocytic Leukemia; NCT04996030: A Study for Oral SY-2101 for Participants With Acute Promyelocytic Leukemia) and one approved (NCT02699723: Arsenic Trioxide and Itraconazole in Treating Patients with Advanced Basal Cell Cancer) clinical trials using arsenic trioxide listed on the NCI Clinical Trials website.

Arsenic and its compounds have been reported to be non-mutagenic. However, DNA methylation, a key epigenetic mechanism that adds a methyl group to the C5 position of cytosine forming 5-methylcytosine, can influence gene expression and subsequently contribute to cancer development [5,6]. It was reported that inorganic arsenic induced global DNA hypomethylation in the livers of mice [7]. It was also reported that inorganic arsenic induced localized DNA hypermethylation or hypomethylation in individual genes in experimental animals [8,9,10,11]. Arsenic induced global DNA hypomethylation by inhibiting the activity of DNA methyltransferase (DNMT) [12,13]. Induction of hypermethylation in the promoter regions of tumor suppressor genes suggests that aberrant DNA methylation may play an important role in arsenic-induced bladder carcinogenesis [14]. Nevertheless, the precise mechanisms by which aberrant DNA methylation contributes to arsenic-induced carcinogenesis remain unclear, primarily due to the lack of suitable experimental models.

The metabolic processing of inorganic arsenic compounds significantly influences their toxic and carcinogenic effects. It is proposed that inorganic arsenic undergoes stepwise metabolism in humans, alternating between the reduction of pentavalent arsenicals to trivalent arsenicals and the oxidative methylation of trivalent arsenicals to pentavalent metabolites [1]. We have focused on dimethylarsinic acid (DMA^V^), a major methylated metabolite of arsenic in most mammals, including humans [1,15,16,17]. DMA^V^ has been shown to induce bladder urothelial carcinoma (UC) in rats when administered in drinking water [18,19] or diet [20]. These studies corroborate epidemiological data that indicate inorganic arsenic is a human bladder carcinogen, and suggest that DMA^V^ may be a relevant component in the carcinogenic risk of inorganic arsenic exposure in humans. IARC categorizes DMA^V^ as Group 2A, indicating probable carcinogenicity to humans. DMA^V^ is non-mutagenic in in vitro tests [1,21]. Our previous study demonstrated that DMA^V^ is not mutagenic in the bladders of *gpt* delta rats [22]. We also found that transplacental DMA^V^ exposure leads to aberrant methylation of histone H3K9, subsequent overexpression of Krt8, and increased cellular proliferation, thereby enhancing lung carcinogenesis in male offspring mice [23]. These findings suggest that epigenetic abnormalities are involved in DMA^V^-induced carcinogenesis. However, the correlation between these genetic alterations and aberrant DNA methylation in DMA^V^-induced bladder carcinogenesis remains to be determined. The present study aims to identify aberrant DNA methylation and understand its impact on gene expression in DMA^V^-induced rat UCs. To discern whether changes in gene expression are early or late events in DMA^V^-induced rat bladder carcinogenesis, we also analyzed gene alterations in the urothelium of rats treated with DMA^V^ for four weeks.

## 2. Materials and Methods

### 2.1. Chemicals and Diets

DMA^V^ (purity > 99%) was purchased from Sigma-Aldrich (St. Louis, MO, USA).

### 2.2. Animals

All animal studies were approved by the Institutional Animal Care and Use Committee of Osaka Metropolitan University Graduate School of Medicine and conducted in accordance with the Guidelines for Proper Conduct of Animal Experiments (Science Council of Japan, 2006). The Laboratory Animal Center of Osaka Metropolitan University Graduate School of Medicine is accredited by the Center for the Accreditation of Laboratory Animal Care and Use (CALAC), Japan Health Sciences Foundation (JHSF), and animal experiments were conducted with reference to the National Research Council’s Guide for the Care and Use of Laboratory Animals and ARRIVE guidelines [24]. Five-week-old male F344/DuCrj rats were obtained from Charles River Japan (Atsugi, Japan). They were housed in plastic cages with hardwood chip bedding in an air-conditioned room at 23 ± 2 °C and 55 ± 5% humidity with a 12 h light/dark cycle and maintained on a basal diet (Oriental MF, Oriental Yeast Co., Tokyo, Japan) and tap water ad libitum. All animals were acclimated for five weeks before being used for experiments.

### 2.3. Animal Experimental Protocols

In the 104-week DMA^V^ bladder carcinogenicity study (#2002BB), 10-week-old F344/DuCrj rats were randomly allocated to groups of 30 rats and were given drinking water containing 0 and 200 ppm DMA^V^ for 104 weeks [25]. Rats that became moribund or survived until the end of week 104 were euthanized by exsanguination under inhalation anesthesia with isoflurane (Abbott Co., Ltd., Tokyo, Japan) using a Small Animal Anesthetizer (Muromachi Kikai Co., Ltd., Tokyo, Japan, MK-A110D) coupled with an Anesthetic Gas Scavenging System (Muromachi Kikai Co., Ltd., MK-T100E), and subjected to laparotomy with excision of the urinary bladder. Macroscopic observation confirmed the presence of five bladder cancers with diameters over 5 mm that are sufficient for DNA extraction and the subsequent DNA methylation assay in the DMA^V^-treatment group. These tumors and five bladder tissues of age-matched control rats were excised, snap-frozen in liquid nitrogen, and stored at −80 °C for microarray gene expression analysis and genome-wide DNA methylation analysis. Total RNA was isolated according to the TRIzol^®^ Reagent’s protocol. Because the object of the present study was to analyze gene expression and DNA methylation in bladder cancers after 104 weeks of exposure, further analysis of tumor incidence was not performed. In addition, to maximize the collection of DNA and RNA, histological examination of the bladder cancers used in the present study was not performed. However, our experience with bladder carcinogenesis is that macroscopic tumors greater than 5 mm in diameter are invariably UCs, including the results of our previous 2-year DMA^V^ bladder carcinogenicity study [18,19].

In the 4-week experiment (#2007AB), 10-week-old F344/DuCrj rats were randomly allocated to groups of 7 rats each and were given drinking water containing 0 and 200 ppm DMA^V^ for 4 weeks [25]. At the end of week 4, the animals were euthanized by exsanguination under inhalation anesthesia as described above and subjected to laparotomy with excision of the urinary bladder. Bladder mucosae were collected from all 7 rats of each group. Briefly, urinary bladders were quickly excised and inverted on wooden applicator sticks. After rinsing with cold RNase-free PBS buffer, bladder epithelial cells were removed by swirling the inverted bladders vigorously in microcentrifuge tubes containing TRIzol^®^ Reagent (Invitrogen, Carlsbad, CA, USA). Total RNA was isolated according to the TRIzol^®^ Reagent’s protocol.

### 2.4. Genome-Wide DNA Methylation Analyses of Rat UCs

Genome-wide DNA methylation analyses were conducted on 5 DMA^V^-induced UCs and 3 normal urinary bladders in the 104-week carcinogenicity study using the reduced representation bisulfite sequencing method (RRBS) by ACTIVE MOTIF (Carlsbad, CA, USA). Briefly, genomic DNA was extracted using the Quick-gDNA MiniPrep kit (Zymo Research, Irvine, CA, USA, D3024) following the manufacturer’s instructions for tissues. The tissues were first Proteinase K digested (0.5% SDS, 0.5 mg/mL PK, 100 mM EDTA, in TE pH 8) rotating at 55 °C overnight; 100 ng of gDNA was digested with TaqaI (NEB, Ipswich, MA, USA, R0149) at 65 °C for 2 h followed by MspI (NEB R0106) at 37 °C overnight. Following enzymatic digestion, samples were used for library generation using the Ovation RRBS Methyl-Seq System (Tecan, Kanagawa, Japan, 0353-32) following the manufacturer’s instructions. In brief, digested DNA was randomly ligated, and, following fragment end repair, bisulfite converted using the EpiTect Fast DNA Bisulfite Kit (Qiagen, Hilden, Germany, 59824) following the Qiagen protocol. After conversion and clean-up, samples were amplified using the Ovation RRBS Methyl-Seq System protocol for library amplification and purification. Libraries were measured using the Agilent 2200 TapeStation System and quantified using the KAPA Library Quant Kit ABI Prism qPCR Mix (Roche, Basel, Switzerland, KK4835). Libraries were sequenced on a NextSeq 500 at SE75. Raw methylation status data were deposited with accession number GSE243372 in the Gene Expression Omnibus (https://www.ncbi.nlm.nih.gov/geo/ (accessed on 16 September 2023)).

### 2.5. Alignment of RRBS Data and Identification of DMRs

The alignment of RRBS data was performed by ACTIVE MOTIF. Briefly, the process of alignment is: First, the Illumina adapter sequence is trimmed from the reads. Then, a custom script is used to trim additional bases that are added during the library creation process to facilitate sequencing. The resulting reads are then mapped to the genome using RRBSMAP using default settings (12 bp seed length, max number of mismatches = 0.08 × read length); only unique alignments are kept (if a fragment maps to more than one location, it is discarded) [26]. Another custom script is then used to remove any PCR duplicates based on a randomized 6-mer barcode; if more than one fragment has the same start and end coordinates as well as the same barcode, all but one are discarded. Alignment data are generated by RRBSMAP. The differential methylation analysis is generated using DMAP [27]. The resulting regions are then annotated using the HOMER software package [28]. Processed data that contain methylation percentages of genes are generated using the software package GBSA 2.0. The data were aligned to the rat (rn6) genome [29]. To identify DMRs (differentially methylated regions), we performed an ANOVA test between the normal bladder tissue (control) and UC samples using DMAP. A heatmap of methylation percentages of “Promoter2K” regions (0–2000 bp upstream of the transcriptional start site) for all samples within the project was generated by the “heatmap.2” function in R packages. Coverage must exceed the minimum (3 CpGs with 3 reads/CpG) for samples to be included. Rows are hierarchically clustered using the Euclidean distance metric. Bright red indicates 0% methylation, black indicates 50% methylation, and bright green indicates 100% methylation. The blue line corresponds to the methylation percentage of the respective regions, thus facilitating visual comparison of methylation percentages across samples (Supplementary Figure S1).

### 2.6. Microarray Gene Expression Analysis

Microarray gene expression analysis of DMA^V^-induced UCs and urothelium from rats treated for 4 weeks with DMA^V^ and their respective controls was performed by Cell Innovator Inc., Fukuoka, Japan, using a GeneChip^®^ Rat Genome 230 2.0 Array. After global median normalization, data cleansing was performed to remove the values for which fluorescence intensity was less than 100. To identify genes with up or downregulated expression, samples from the DMA^V^ groups and their respective controls were compared, and Z-scores and ratios (non-log scale fold-change) were calculated from the normalized signal intensity of each probe. We then established the criteria for regulated genes: (upregulated genes) Z score ≥ 2.0 and ratio ≥ twofold; (downregulated genes) Z score ≤ −2.0 and ratio ≤ 0.5. Raw expression data were deposited with accession number GSE207963 for the 4-week experiment and GSE207973 for DMA^V^-induced UCs in the Gene Expression Omnibus (https://www.ncbi.nlm.nih.gov/geo/).

### 2.7. Quantitative Real-Time PCR (qPCR)

cDNA synthesis was performed with 1 µg of RNA using SuperScript IV VILO Master Mix (Thermo Fisher Scientific K.K.). Primers and probes (Taqman Gene Expression Assay) for rat Cpxm1 (Rn01484602_g1), rat Opcml (Rn00587759_m1), rat Kcnd3 (Rn04339183_m1), rat Tbx20 (Rn01524829_m1), rat 18s (4310893E), human CPXM1 (Hs00219709_m1), and human RPLP2 (Hs01115130_g1) were purchased from Thermo Fisher Scientific, MA, USA. qPCR assays were carried out with the Applied Biosystems 7500 Fast real-time PCR machine (Applied Biosystems, Foster City, CA, USA). Values for target genes were normalized to those for rat 18s or human RPLP2 using the comparative Ct method.

### 2.8. Correlations between CPXM1 Promoter Methylation and mRNA Expression Levels in Human UCs

The MEXPRESS is a web-based tool designed for the integration and visualization of expression, DNA methylation, and clinical TCGA data at a single-gene level (http://mexpress.be (accessed on 5 December 2022)) [30,31]. Pearson correlation analysis was used to compare expression and methylation data [30]. By utilizing this tool, we investigated the correlations between *CPXM1* promoter methylation and its corresponding mRNA expression levels in 450 human bladder UCs.

### 2.9. Examination of the DNA Methylation Status within the CPXM1 Promoter Region in Human UC Cell Lines

Genomic structures of *CPXM1* were obtained from the UCSC genome browser including a chromosomal ideogram. The transcriptional start site (TSS) of *CPXM1* is referenced from the NCBI database (NC_000020, 2800627). The location of the TSS was also confirmed using the KERO database (http://kero.hgc.jp/ (accessed on 24 September 2023)). The black arrows depict CpG sites wherein the expression of genes and the status of DNA methylation manifest an inverse correlation in cases of human UCs, as disclosed by MEXPRESS. Methylation levels at CpG sites correlated by MEXPRESS in human UC cell lines are illustrated. The X-axis shows the MAPINFO for each CpG site. The Y-axis shows the methylation levels of each CpG site. Data on the methylation status of human UC cell lines T24, UMUC3, TCCSUP, 5637, HT1376 were obtained from the GEO database (Platform GPL 13534).

### 2.10. Effects of DNA Methyltransferase Inhibitor in Human UC Cell Lines

The human UC cell lines T24 and UMUC3 were purchased from the American Type Culture Collection. Cells were grown in DMEM (FUJIFILM Wako Pure Chemical Corporation, Tokyo, Japan) supplemented with 10% FBS (GE Healthcare Japan) and were kept at 37 °C in a 5% CO_2_ atmosphere. On the first day (day 0), cells were seeded. The medium was then replaced on days 1 and 3 with medium containing 0 or 1.0 μM DNA methyltransferase inhibitor 5-aza-2′-deoxycitidine (5-aza-dC, FUJIFILM Wako Pure Chemicals Corporation). On day 4, the cells were harvested for RNA expression analysis. Total RNA was extracted from cell lines using the RNeasy Mini kit (QIAGEN) according to the manufacturer’s instructions.

### 2.11. Immunohistochemical Analysis

A tissue microarray containing 160 cases of human UCs and 11 cases of normal bladder tissue (BL2081, US Biomax, Inc., Rockville, MD, USA) was examined for the expression of CPXM1 by immunohistochemical staining using the avidin–biotin–peroxidase complex (ABC) method. The section was deparaffinized and dehydrated, followed by antigen retrieval at 98 °C for 20 min in Tris-EDTA buffer (pH 8.0). Endogenous peroxidase activity was blocked with 3% H_2_O_2_ in distilled water for 5 min. After blocking nonspecific binding with goat serum at 37 °C for 15 min, the section was incubated with primary antibodies for CPXM1 (Invitrogen, #PA-5-31386, 1:1000) overnight at 4 °C. Immunoreactivity was detected using VETASTAIN Elite ABC kit for rabbit (Vector Laboratories, Burlingame, CA, USA, Rabbit IgG, #PK-6101) and 3,3′-diaminobenzidine tetrahydrochloride (Dojindo Molecular Technologies, Kumamoto, Janpan). CPXM1 was expressed in the cytoplasm of normal urothelial cells. All carcinoma specimens showed less than 5% or more than 90% positive staining of CPXM1 in the cytoplasm of the carcinoma cells, therefore, specimens were defined as CPXM1 positive (CPXM1^posi^) when more than 90% of the carcinoma cells showed positive staining and CPXM1 negative (CPXM1^Neg^) when less than 5% of the carcinoma cells showed positive staining. Evaluation was performed by two pathologists (M. Gi and T. Yamamoto) separately. Discrepant cases were studied together by the two pathologists and a consensus was reached.

### 2.12. Statistical Analysis

Mean values in gene expression analyses are reported as mean ± SD. Statistical analysis was performed using GraphPad Prism version 9 software (GraphPad Software). Homogeneity of variance was tested by the F-test. Differences in mean values of mRNA expression between the control and the treated samples were evaluated by 2-tailed Student’s *t*-test when variance was homogeneous and 2-tailed Mann–Whitney test when variance was heterogeneous (two-group comparisons).

## 3. Results

### 3.1. Determination of Aberrant DNA Methylation in DMA^V^-Induced Rat UCs

Genome-wide DNA methylation patterns in DMA^V^-induced rat UCs and the normal bladders of male F344 rats were determined using RRBS. Hierarchical clustering analysis of genome-wide DNA methylation status revealed that the hierarchical dendrogram was divided into two main clusters: Cluster A, which comprised one UC and three normal bladders, and Cluster B, which comprised four UCs (Supplementary Figure S1). Notably, Cluster A was further separated into two sub-clusters: one consisting of one UC and the other of three normal bladders. This suggests that the genome-wide DNA methylation patterns between DMA^V^-induced UCs and normal bladders are distinct. From bioinformatics analysis, differentially methylated regions (DMRs) were detected in 166,670 regions. There were 399 regions that were located on CpG islands, with distance to the transcriptional start site (TSS) from −500 to +100, and with methylation level differences of more than 10% between the UCs and normal bladder tissue. Additionally, there were 246 genes whose promoter regions were highly methylated in all five UCs compared to all three normal bladder urothelium samples (Supplementary Table S1). These genes are considered representative of the hypermethylated genes in DMA^V^-induced UCs.

### 3.2. Determination of Genes Regulated by DNA Hypermethylation in DMA^V^-Induced Rat UCs

**Figure 1:**
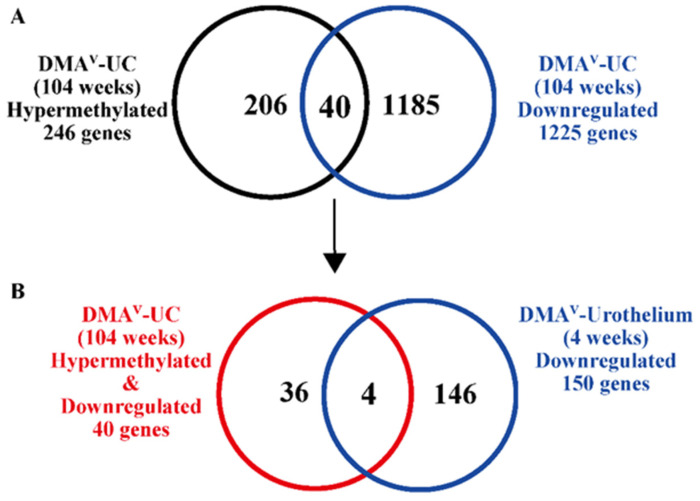
DNA methylation alterations and downregulated genes in DMA^V^-induced rat bladder carcinogenesis. (**A**) Numbers of hypermethylated (246) and downregulated (1225) genes in DMA^V^-induced UCs. Forty genes were both hypermethylated and downregulated in all 5 rat UCs. (**B**) A total of 150 genes were downregulated in the urothelium of rats treated with DMA^V^ for four weeks. Four of these genes were also both hypermethylated and downregulated in the 5 UCs (**A**).

To identify genes potentially regulated by DNA hypermethylation, we conducted a microarray gene expression analysis of the five DMA^V^-induced UCs, which were also analyzed for methylation status as described above. A total of 1225 genes were downregulated in all DMA^V^-induced UCs compared to the normal bladder urothelium in the five control rats. Notably, by aligning the profiles of the 246 hypermethylated genes with the 1225 downregulated genes (Figure 1A), we found that 40 of these downregulated genes were also hypermethylated in all of the rat UCs (Table 1). This strongly suggests that these 40 genes were the targets of hypermethylation and downregulated due to DNA hypermethylation. Functional categorization using IPA software indicated that this subset of 40 genes mainly encompassed transcriptional regulators, transmembrane receptors, peptidases, enzymes, transporters, and ion channels.

### 3.3. Evaluation of Gene Expression of Hypermethylated and Downregulated Genes in DMA^V^-Induced UCs in the Early Stage of DMA^V^-Induced Bladder Carcinogenesis

**Figure 2:**
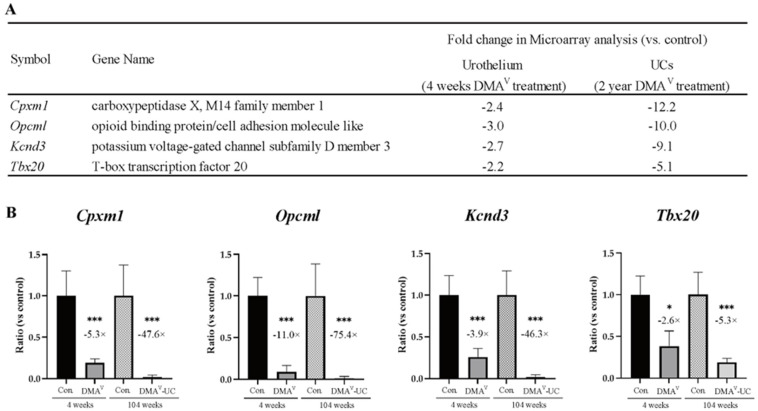
(**A**) Microarray gene expression data of four genes (Cpxm1, Opcml, Kcnd3, and Tbx20) with downregulated mRNA expression in both the urothelium of rats treated with DMA^V^ for 4 weeks and in DMA^V^-induced rat UCs. (**B**) qPCR mRNA expression data of Cpxm1, Opcml, Kcnd3, and Tbx20 in the urothelium of rats treated with DMA^V^ for 4 weeks and in DMA^V^-induced rat UCs (5 samples/group). Significant differences from the respective controls at * *p* < 0.05 and *** *p* < 0.001.

To identify genes that could contribute to the onset of DMA^V^-induced bladder carcinogenesis, we conducted a microarray gene expression analysis of the bladder urothelium of rats treated with DMA^V^ for 4 weeks. From this analysis, we selected genes that were both downregulated in the DMA^V^-treated urothelium and were among the 40 hypermethylated and downregulated genes identified in the UCs described above. Four genes—carboxypeptidase X, M14 family member1(*Cpxm1*), opioid binding protein/cell adhesion molecule like (*Opcml*), potassium voltage-gated channel subfamily D member 3(*Kcnd3*), and T-box transcription factor 20(*Tbx20*)—were identified as being notably downregulated in the urothelium of rats treated for four weeks with DMA^V^ and as being hypermethylated and downregulated in the UCs (Figure 1B). Intriguingly, microarray data revealed that the mRNA expression levels of these four genes decreased by 2- to 3-fold in the urothelium treated with DMA^V^ for four weeks and this reduction was more pronounced in the UCs, with a 5- to 10-fold decrease compared to their respective controls (Figure 2A). Quantitative PCR corroborated the microarray findings (Figure 2B). In the DMA^V^-treated urothelium, expression of *Cpxm1* decreased 5.3-fold, whereas in the UCs it dropped by 47.6-fold. Similarly, expression of *Opcml* declined 11.0-fold in the DMA^V^-treated urothelium and 75.4-fold in the UCs. Expression of *Kcnd3* fell 3.9-fold in the DMA^V^-treated urothelium and 46.3-fold in the UCs. Lastly, expression of *Tbx20* was reduced 2.6-fold in the DMA^V^-treated urothelium and 5.3-fold in the UCs. These findings suggest that these four genes are likely downregulated due to hypermethylation and may play critical roles from the early to later stages of DMA^V^-induced rat bladder carcinogenesis. In the current study, we focused on *Cpxm1*, a peptidase involved in peptide metabolism and protein processing. While its role in cancer remains largely undefined, *CPXM1* has been reported to be hypermethylated and downregulated in breast cancer [32]. Importantly, to the best of our knowledge, no information regarding the role of *CPXM1* in the UCs is available in the literature.

### 3.4. Correlation between Methylation Status and mRNA Expression Level of CPXM1 in Human UCs

**Figure 3:**
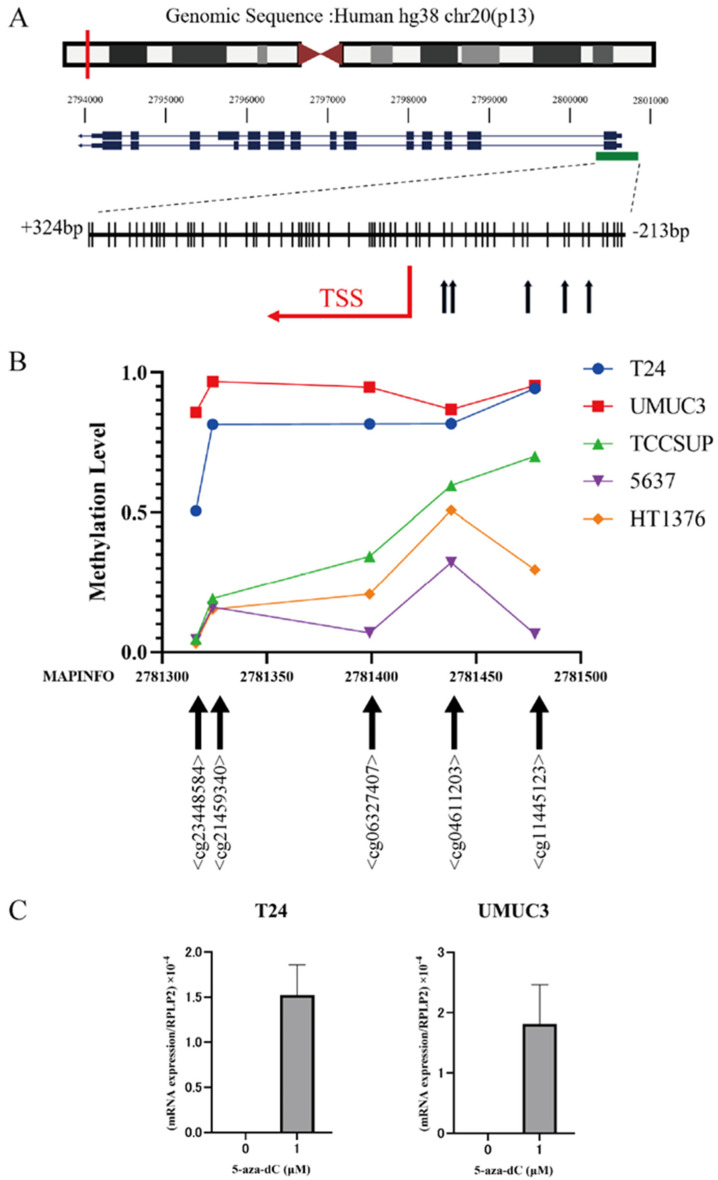
Methylation status of *CPXM1* in human UC cell lines. (**A**) Genomic locations of *CPXM1* from the UCSC genome browser including chromosomal ideogram, RefSeq gene map (blue lines and bars), and CpG island (green bar). TSS of *CPXM1* (red arrow). The red line depicts the location of CPXM1 in the chromosome 20. The black arrows depict CpG sites wherein the mRNA expression and the status of DNA methylation are negatively correlated in human UCs using MEXPRESS. (**B**) The X-axis shows the MAPINFO for each CpG site. The Y-axis shows the methylation rate of each CpG site. (**C**) After 5-aza-dC treatment, mRNA expression of *CPXM1* was significantly restored in T24 and UMUC3.

To investigate the relationship between DNA methylation status and *CPXM1* expression in human UCs, we utilized the MEXPRESS database. Our analysis of a cohort comprising 450 human UC samples stored in the MEXPRESS database revealed a negative correlation between the mRNA expression levels of *CPXM1* and the methylation status of five CpG sites in its promoter region (Figure 3A; Supplementary Figure S2).

### 3.5. The Correlation Analysis between CPXM1 Expression and DNA Methylation Level in Human UC Cells

Methylation data for five human UC cell lines (T24, UMUC3, TCCSUP, 5637, and HT1376) were obtained from the Gene Expression Omnibus (GEO; Platform GPL13534). Based on the methylation status of five CpG sites in the CPXM1 promoter, previously identified as hypermethylated and negatively correlated with CPXM1 expression in human UCs (Figure 3A; Supplementary Figure S2), the six cell lines were broadly divided into two groups based on their methylation status. The first group, comprising T24 and UMUC3 showed higher levels of hypermethylation (Figure 3B). The second group, consisting of TCCSUP, HT1376, and 5637, exhibited lower levels of hypermethylation.

Therefore, we selected cell lines T24 and UMUC3 for DNA demethylation analysis using 5-aza-dC. No mRNA expression of CPXM1 was observed in T24 and UMUC3 in the absence of 5-aza-dC treatment. However, treatment with 5-aza-dC markedly restored the expression of CPXM1 (Figure 3C). These findings suggest that CPXM1 expression is effectively silenced through hypermethylation in these human UC cell lines.

### 3.6. Protein Expression of CPXM1 in Human UCs

**Figure 4:**
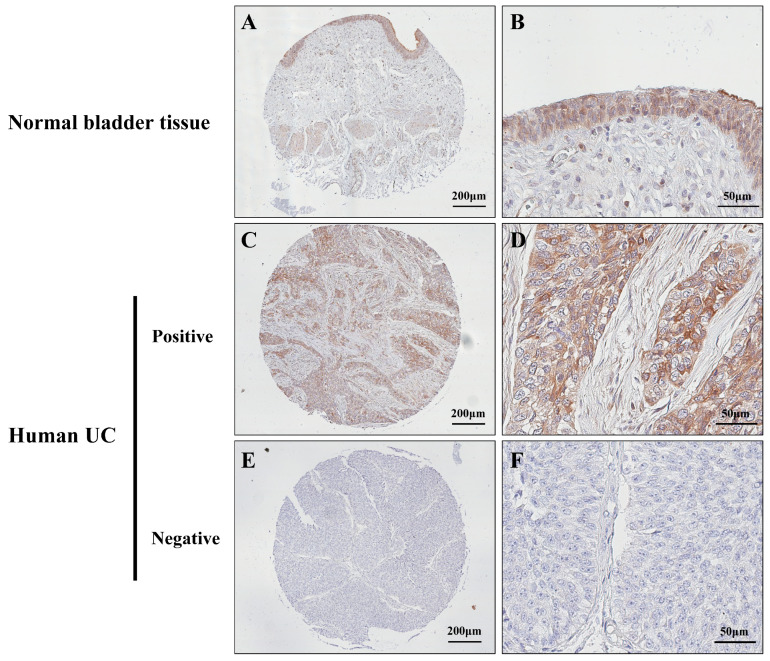
Immunohistochemistry of CPXM1 in human UCs. (**A**) CPXM1 was positive in the urothelium of normal bladder tissue. (**B**) High magnification of (**A**). (**C**) A CPXM1posi human UC. (**D**) High magnification of (**C**). (**E**) A CPXM1nega human UC. (**F**) High magnification of (**E**).

The expression of CPXM1 in human UCs was investigated using a high-density tissue array comprising 160 UC samples and 11 normal bladder tissues (Table 2). In normal bladder tissue, CPXM1 was positively expressed in the cytoplasm of the urothelial cells (Figure 4A,B). All normal bladder tissues were positive for CPXM1 expression (11/11, 100%). In contrast, only a small fraction of the UC samples showed positive CPXM1 expression (23/160, 14%) (Figure 4C,D), while the majority were negative for CPXM1 (137/160, 86%) (Figure 4E,F). The expression of CPXM1 was significantly reduced in the UCs compared to the normal bladder tissue. No significant difference was observed in the incidence of CPXM1-positive expression between the UCs of T1 and T2 or higher grades of UC.

## 4. Discussion

The current study revealed that aberrant DNA methylation plays a role in DMA^V^-induced rat bladder carcinogenesis. To the best of our knowledge, this is the first study to identify aberrant DNA methylation in arsenic-induced rat bladder carcinogenesis.

We identified 40 genes that were downregulated and displayed hypermethylation in their promoter regions in DMA^V^-induced rat UCs. These genes primarily include transcriptional regulators, transmembrane receptors, peptidases, enzymes, transporters, and ion channels. This suggests that changes in DNA methylation might affect multiple pathways, contributing to DMA^V^-induced rat bladder carcinogenesis. It is established that inorganic arsenic can cause aberrant DNA methylation by inhibiting DNMT activity [12,13]. Additionally, the depletion of S-adenosylmethionine (SAM), which serves as a methyl donor during the metabolism of inorganic arsenic, is also believed to lead to aberrant DNA methylation [33,34,35]. Given that DMA^V^ is a urinary metabolite of inorganic arsenic, these mechanisms could underlie the aberrant DNA methylation observed in DMA^V^-induced bladder carcinogenesis. However, the precise mechanisms through which DMA^V^ induces aberrant DNA methylation remains unclear.

To investigate genes implicated in the early stages of carcinogenesis, we analyzed gene expression in rat bladder urothelium following a 4-week DMA^V^ treatment. We identified Cpxm1, Opcml, Kcnd3, and Tbx20 as genes downregulated in the early phase of DMA^V^-induced bladder carcinogenesis.

*CPXM1*, also termed carboxypeptidase X-1 (*CPX-1*), stands out as an atypical member of the carboxypeptidase family. Despite its classification, it lacks catalytic activity and functions as a secreted collagen-binding glycoprotein [36,37]. In mice, *Cpxm1* plays a pivotal role in the transition from pre-osteoclasts to mature osteoclasts [38]. Furthermore, it is posited to act as a positive regulator of adipogenesis by influencing extracellular matrix remodeling [39]. Its specific function in cancer remains elusive. A study exploring epigenetic markers of breast cancer—utilizing breast cancer tissues, breast cancer cell lines, and blood samples from healthy participants—proposed that the expression of *CPXM1* is epigenetically controlled, potentially functioning as a tumor suppressor gene in breast cancer cells [32].

We found an inverse relationship between *CPXM1* mRNA expression levels and the methylation status of its promoter region in human UCs. This led us to investigate whether *CPXM1*’s expression might be influenced by promoter region hypermethylation in human UCs. In cells like T24 and UMUC3, where the *CPXM1* promoter region was prominently methylated, mRNA expression of *CPXM1* was notably revived upon treatment with DNA methyltransferase inhibitors. Moreover, tissue microarray outcomes from human UCs indicated a pronounced reduction in *CPXM1* expression in UCs, irrespective of T-stage, tumor grade, or gender. These data indicate that *CPXM1*’s expression in human UCs may be suppressed due to DNA hypermethylation.

An elevated methylation status in the *CPXM1* promoter region in breast cancer tissues relative to normal tissues has been reported, and elevated *CPXM1* expression has been linked with extended survival [40]. A prognostic index built from a trio of genes, inclusive of *CPXM1*, correlated with responsiveness to immune checkpoint inhibitors (ICI) in head and neck squamous cell carcinoma [41]. In the context of melanoma, a gene panel encompassing *CPXM1* was discerned as a predictive tool for anti-PD-1 therapy response [42]. Although these reports do not delve into the intricate role of *CPXM1* in cancer, epidemiological findings suggest its expression in malignancies is correlated with prognosis and ICI responsiveness. Recent studies affirm the efficacy of ICI in treating UCs [43,44,45], positioning *CPXM1* as a potential marker for prognosis or treatment efficacy in human UCs. The fact that *CPXM1* was downregulated as early as 4 weeks after DMA^V^ treatment suggests that *CPXM1* might serve as an early diagnostic marker or a therapeutic target for arsenic-induced human UCs. It should be noted that a limitation of the present study is the absence of data regarding the exact function of CPXM1 in human UCs. These data are crucial for any potential clinical application of this gene, both as a therapeutic target and for early diagnosis.

*KCND3*, *OPCML*, and *TBX20* were all hypermethylated in DMA^V^-induced UCs and exhibited downregulation from the onset of bladder carcinogenesis to the progression of UC. The role of *KCND3* in cancers remains unclear. Epigenetic silencing of *OPCML* has been reported in a spectrum of human malignancies, including human UC, and loss of *OPCML* could spur cancer development by attenuating intercellular adhesion [46,47,48,49]. *TBX20*’s expression is curtailed due to hypermethylation of its promoter region in colon cancer, where it acts as a tumor suppressor. Its mechanism of action is believed to be via the inhibition of NHEJ-mediated DNA repair [50]. These observations are in general agreement with the premise that the genes identified in this study have a role in both the early and later stages of arsenic-induced bladder carcinogenesis.

## 5. Conclusions

Our findings demonstrate that aberrant DNA methylation in DMA^V^-induced bladder carcinogenesis in rats occurred in the early stage of arsenic-induced bladder carcinogenesis, and that the resulting downregulation of expression of particular genes could have a role in transformation and tumor development. Further studies to evaluate the functions of CPXM1 and other genes that undergo aberrant DNA methylation will advance our understanding of the role of aberrant DNA methylation in arsenic bladder carcinogenesis and will also facilitate the identification of novel therapeutic targets for arsenic-related bladder cancers.

## Figures and Tables

**Table 1:** Forty genes hypermethylated and downregulated in DMA^V^-induced UCs.

Gene Symbol	Gene Name	Fold Change	Function
*Tbx20*	T-box transcription factor 20	−5.1	transcription regulator
*Pcbp3*	poly(rC) binding protein 3	−3.1	transcription regulator
*Pitx2*	paired like homeodomain 2	−2.1	transcription regulator
*Foxe3*	forkhead box E3	−2	transcription regulator
*Prrx2*	paired related homeobox 2	−1.6	transcription regulator
*Scara3*	scavenger receptor class A member 3	−6.5	transmembrane receptor
*Sema5a*	semaphorin 5A	−2.2	transmembrane receptor
*Robo2*	roundabout guidance receptor 2	−1.5	transmembrane receptor
*Cpxm1*	carboxypeptidase X, M14 family member 1	−12.2	peptidase
*Ctsz*	cathepsin Z	−3	peptidase
*Mmp23bB*	matrix metallopeptidase 23B	−2.9	peptidase
*Gpx7*	glutathione peroxidase 7	−1.7	enzyme
*Polg*	DNA polymerase gamma, catalytic subunit	−1.7	enzyme
*Mpst*	mercaptopyruvate sulfurtransferase	−1.7	enzyme
*Nmnat2*	nicotinamide nucleotide adenylyltransferase 2	−1.6	enzyme
*Cyp7b1*	cytochrome P450 family 7 subfamily B member 1	−1.6	enzyme
*Pde8a*	phosphodiesterase 8A	−1.5	enzyme
*Pclo*	piccolo presynaptic cytomatrix protein	−3.1	transporter
*Atp2a3*	ATPase sarcoplasmic/endoplasmic reticulum Ca^2+^ transporting 3	−1.6	transporter
*Kcnd3*	potassium voltage-gated channel subfamily D member 3	−9.1	ion channel
*Kcnq5*	potassium voltage-gated channel subfamily Q member 5	−3.5	ion channel
*Cacna2d1*	calcium voltage-gated channel auxiliary subunit alpha2delta 1	−2.9	ion channel
*Kcne5*	potassium voltage-gated channel subfamily E regulatory subunit 5	−2.3	ion channel
*Cacna1bB*	calcium voltage-gated channel subunit alpha1 B	−1.8	ion channel
*Hcn1*	hyperpolarization activated cyclic nucleotide gated potassium channel 1	−1.7	ion channel
*Asic2*	acid sensing ion channel subunit 2	−1.6	ion channel
*Ptprm*	protein tyrosine phosphatase receptor type M	−3.6	phosphatase
*Npr2*	natriuretic peptide receptor 2	−2.6	G-protein coupled receptor
*Prkcb*	protein kinase C beta	−1.9	kinase
*Des*	desmin	−40.9	other
*Opcml*	opioid binding protein/cell adhesion molecule like	−10	other
*Clmp*	CXADR like membrane protein	−4.1	other
*Brinp2*	BMP/retinoic acid inducible neural specific 2	−3.1	other
*Stxbp6*	syntaxin binding protein 6	−3.1	other
*Fstl1*	follistatin like 1	−2.7	other
*Macroh2a2*	macroH2A.2 histone	−2.1	other
*Sprn*	shadow of prion protein	−1.9	other
*Arhgef25*	Rho guanine nucleotide exchange factor 25	−1.8	other
*Mfap2*	microfibril associated protein 2	−1.7	other
*Coro1a*	coronin 1A	−1.6	other

**Table 2:** CPXM1 expression in human UCs.

	n	Expression		*p*-Value
		Negative	Positive	
NBT	11	0	11	*p* < 0.001
		(0%)	(100%)	
UCs	160	137	23	
		(86%)	(14%)	
T-stage				
T1	50	44	6	0.56
		(88%)	(12%)	
>T2	110	93	17	
		(85%)	(15%)	
Gender				
male	125	107	18	0.98
		(86%)	(14%)	
female	35	30	5	
		(86%)	(14%)	
Tumor grade				
HG	110	96	14	0.37
		(87%)	(13%)	
LG	50	41	9	
		(82%)	(18%)	

NBT: Normal bladder tissue, UC: Urothelial carcinoma, HG: High-grade urothelial carcinoma, LG: Low-grade urothelial carcinoma.

## Data Availability

The data presented in this study were uploaded to the Gene Expression Omnibus (GEO) repository, reference numbers: GSE243372, GSE207963, and GSE207973.

## Supplementary Materials

The following supporting information can be downloaded at: https://www.mdpi.com/article/10.3390/cancers15215274/s1, Figure S1: DNA methylation analysis of rat DMA^V^-induced UCs; Figure S2: The correlation between mRNA expression of CPXM1 and its DNA methylation shown using MEXPRESS; Table S1: A total of 246 genes hypermethylated and downregulated in DMA^V^-induced UCs.
