# Supplementary material for: DNA Methylation Aberrations in Dimethylarsinic Acid-Induced Bladder Carcinogenesis
Source: Cancers (Basel). 2023 Nov 3;15(21):5274. doi: 10.3390/cancers15215274 (PMC10648661; doi:10.3390/cancers15215274)
Supplement: Supplementary file 1 [file cancers-15-05274-s001.zip › Supplementary Figure 1 and 2 .pdf]

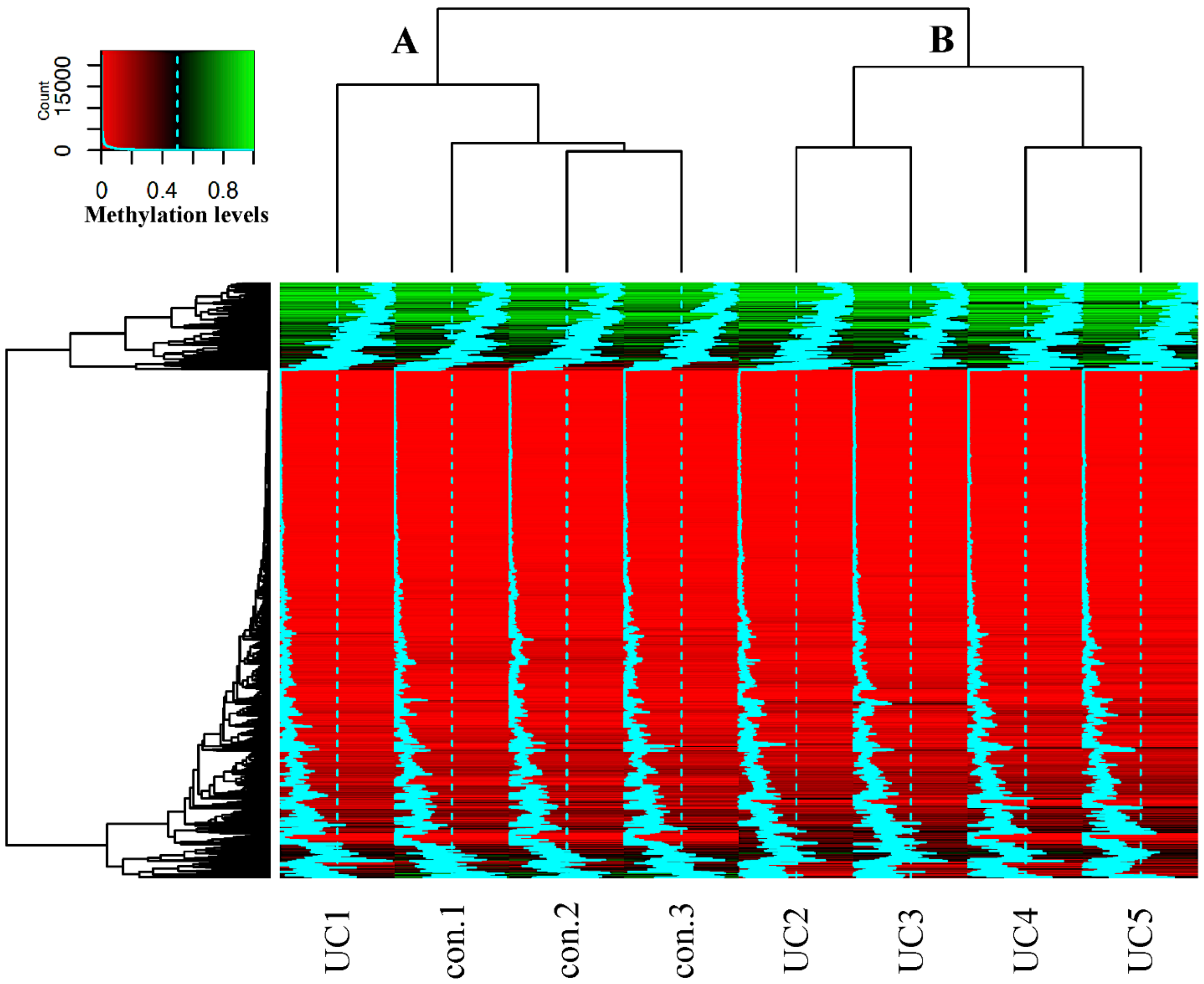

Supplementary Figure S1.

DNA methylation analysis of rat DMA^{V}-induced UCs

A heat map of methylation profiles of UCs and normal bladder tissue of the control group (control) with a hierarchical clustering process. Bright red indicates 0% methylation, black indicates 50% methylation, and bright green indicates 100% methylation. The blue line corresponds to the methylation levels of the respective regions, and the dotted line represents 50% methylation.

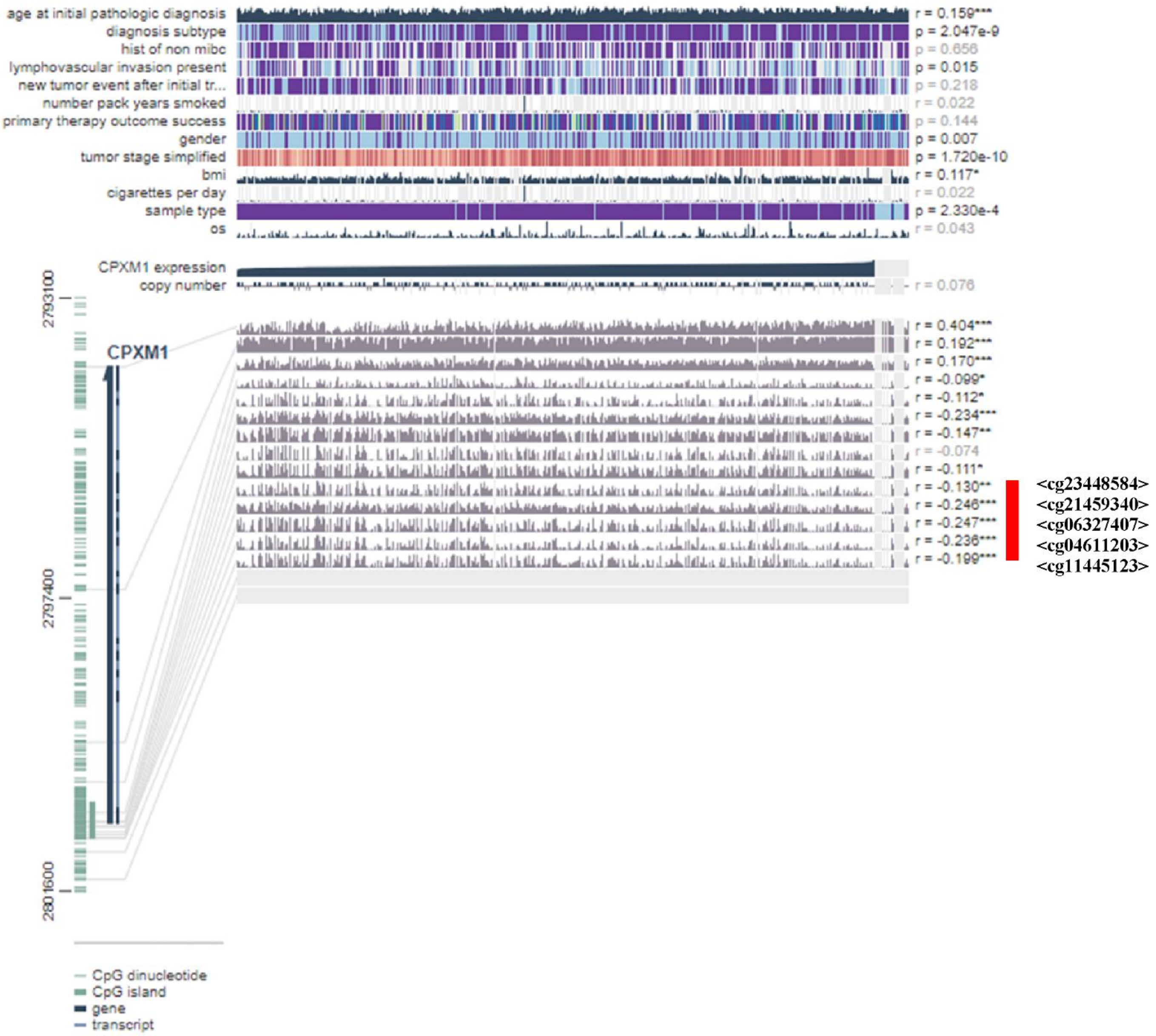

Supplementary Figure S2.

The correlation between mRNA *CPXM1* expression and its DNA methylation was analysed using MEXPRESS in 450 human bladder UCs. The red bar indicates the regions that mRNA expression of *CPXM1* and its DNA methylation level in the promoter region are negatively correlated. (* $p < 0.05$, ** $p < 0.01$, *** $p < 0.001$)
